# Postural Responses in Trauma-Experienced Individuals

**DOI:** 10.3390/biomedicines12122766

**Published:** 2024-12-04

**Authors:** Adam Koncz, Dora Egri, Mustafa Yildirim, Anna Lobko, Evelin Máté, Jennifer W. McVige, Kristof Schwartz

**Affiliations:** 1 Institute of Health Promotion and Sport Sciences, ELTE Eötvös Loránd University, Bogdánfy Street 12, 1117 Budapest, Hungary; koncz.adam@ppk.elte.hu (A.K.);; 2 Department of Health and Nursing Sciences, Faculty of Health and Sport Sciences, Széchenyi István University, Egyetem tér 1, 9026 Győr, Hungary; 3 Department of Neurology, Kaplan Medical Center, Derech Pasternak 1, Rehovot 76100, Israel; 4 DENT Neurologic Institute, 3980 Sheridan Dr, Amherst, NY 14226, USA

**Keywords:** adverse childhood experiences, balance, posture, valance, arousal

## Abstract

**Background:** Balance and proprioception are essential elements in postural control and injury prevention. Proprioception, the body’s sense of position and movement, is closely tied to balance, which depends on input from the visual, vestibular, and somatosensory systems. This article explores the link between trauma experiences and proprioceptive dysfunction, emphasizing how heightened muscle tone, dissociation, and altered sensory processing contribute to balance issues and the risk of injury. **Method:** The study included 48 participants, aged 18–25. Participants completed the Emotional Regulation Scale, Dissociative Experiences Scale II, and Childhood Trauma Questionnaire, after which they had to stand on a BTrackS Balance Plate while being exposed to images that are designed to evoke emotions from the OASIS image set. The balance plate software calculated outcomes of the participants’ postural sway (total sway, sway area, root mean square (RMS) to the mediolateral (ML) and anteroposterior (AP) way, and excursion to ML and AP ways). **Results:** Dissociative experience shows significant correlation with RMS ML when viewing positive pictures (rτ = 0.207, *p* = 0.045) and when viewing negative pictures again; scores with RMS ML (rτ = 0.204, *p* = 0.049) but also with RMS AP (rτ = 0.209, *p* = 0.042) and with Excursion ML (rτ = 0.200, *p* = 0.049) were significant. Experiences of physical abuse affected certain indicators of postural sway when viewing positive images compared to participants with no such experience (sway area: U = 374.50, *p* = 0.027; RMS AP: U = 383.50, *p* = 0.016; Excursion ML: U = 397.00, *p* = 0.007). Similarly, physical neglect affected postural sway during viewing of negative images (sway area: U = 366.50, *p* = 0.003; RMS AP: U = 371.00, *p* = 0.004; Excursion ML: U = 347.00, *p* = 0.034; and Excursion AP: U = 353.00, *p* = 0.010). **Conclusions:** The study highlights that dissociation disrupts balance in trauma survivors, especially under emotional stress which highlights the potential for motor-based treatments.

## 1. Introduction

Balance and proprioception are essential aspects of human motor control, crucial for maintaining postural stability and preventing injuries during daily activities. Proprioception is the body’s ability to sense the position of its parts in space, facilitated by sensory receptors in muscles, joints, and tendons. The proprioceptive system continuously relays information to the brain about body position and movement, ensuring smooth, coordinated motion. Integrating sensory input from the vestibular system, vision, and proprioception helps keep the body’s center of mass stable over its base of support [1]. Studies have shown that proprioceptive training can significantly improve balance, with *p*-values of <0.05 which indicate that changes are statistically significant. This suggests that the role of proprioceptive feedback in maintaining stability and preventing falls is crucial [2]. The interdependent relationship between balance and proprioception allows even the most mundane motor activities, such as walking or reaching, to be executed with precision and in a controlled manner [3].

Improving balance and proprioception is particularly vital in sports since the demands on the musculoskeletal system are high and the risk of injury is elevated [4]. Greater ankle proprioception has been linked to enhanced athletic performance and a reduced risk of lower limb injuries. This enables athletes to have better control and stability, which are very important to prevent possible physical traumas during challenging movements by integrating proprioceptive input with other sensory afferent inputs [5]. Severe complications from falls include fractures, head injuries, and disablement; thus, the need for good balance and intact proprioception is undoubted. Maintaining these physical abilities is crucial for preventing falls and ensuring mobility. Rehabilitation programs focusing on balance and proprioceptive training have proven effective in reducing fall risk [6], highlighting their clinical importance in both injury prevention and recovery.

Apart from aging and physical injuries, balance and proprioception can also be affected by psychological factors, particularly stress and trauma [1]. Emerging research suggests that the mind–body connection is critical in maintaining balance. One area of growing interest is the impact of Adverse Childhood Experiences (ACEs) on balance and proprioceptive function. ACEs include several forms of traumatic events, such as physical, emotional, and sexual abuse, neglect, and household dysfunction [7]. Research shows that nearly 61% of adults have experienced at least one ACE, with about 16% experiencing four or more, placing them at increased risk for numerous physical and mental health issues [8]. While the psychological outcomes of ACEs are greatly documented, including symptoms like depression, anxiety, and PTSD, much of the physical consequences remain unclear. This is especially true regarding such systems as proprioception and balance that can potentially make one vulnerable to injury later in life.

Trauma essentially affects these systems through the body’s response to stress. The autonomic nervous system mediates the response to stress in the form of the fight-or-flight response to perceived threats of harm [9]. More recently, research has emphasized the “freeze” response, where an individual, feeling overwhelmed, becomes immobilized [10]. This response is dominated by states of increased arousal with increased muscle tension and may lead to disrupted motor control and distorted proprioception [11]. Sustained hypervigilance and muscle rigidity in trauma survivors may adversely affect proprioceptive feedback and balance [12]. In addition, emotional arousal, such as in the form of fear or anxiety, is associated with increased muscle tone that may adversely affect fine motor and postural stability [13].

Stress triggers various physiological and behavioral responses, one of which is the freezing reaction, a defense mechanism characterized by immobilization in the face of perceived threats. While this response is evolutionarily adaptive, chronic stress and repeated freezing episodes may have detrimental effects on the musculoskeletal system. Prolonged muscle tension, restricted movement, and dysregulation of motor control during freezing can increase the risk of musculoskeletal injuries. Understanding the interplay between stress, the freezing response, and injury prevalence is crucial for developing preventive strategies and therapeutic interventions aimed at mitigating the long-term physical consequences of stress-induced motor inhibition.

Post-Traumatic Stress Disorder (PTSD), a common illness that can occur after experiencing trauma, is characterized by symptoms like hypervigilance, emotional numbness, and flashbacks [14]. Data from the National Center for PTSD show that approximately 7 to 8% of individuals experience PTSD at some point in their lives [15]. While the psychological effects of PTSD are rather well recognized, the physical manifestations, particularly as they pertain to balance and proprioception, are for the most part uncharted [16]. Recent studies suggest that individuals with PTSD experience increased muscle tone, which may protect against perceived threats but also interfere with normal movement and proprioceptive awareness [17]. It has been shown that up to 50% of people with PTSD report having chronic pain which is potentially linked to muscle hypertonicity [18]. This in turn might be part of a positive feedback loop perpetuating symptoms of dissociation wherein the trauma survivor feels disconnected from his/her body, which further impairs the proprioceptive function and enhances the risk of falls and injuries [19]. 

Research has shown that individuals suffering from PTSD commonly experience a sense of bodily rigidity, which may serve as a protective mechanism from manipulations coming from the outside, such as those tested in experiments like the rubber hand illusion [20]. A possible negative correlation has been identified between the strength of this illusion and the sense of bodily autonomy in participants with PTSD. This finding highlights the relationship between PTSD, dissociative symptoms, and proprioceptive experiences [17]. Given such results, a relationship between body perception in PTSD individuals and their responses to external manipulations, it is relevant to investigate how this may be connected to muscle tension and the general stress response, providing insights into the physiological mechanisms that contribute to trauma-related physical symptoms.

Despite its potential to impact motor control, remarkably few studies have investigated the relationship between PTSD and impairments in balance and proprioception. Some evidence has suggested that stress and anxiety may serve as detrimental factors to postural stability [12]. Acute stress has been shown to increase body sway, indicating impaired postural control. Despite this, there remains a notable gap in the literature regarding the specific ways in which proprioceptive dysfunction may contribute to balance issues in individuals with trauma histories, including those diagnosed with PTSD. Given the significant role that proprioception and balance play in preventing injuries, this area warrants further investigation [21].

This paper discusses the connection between adverse traumatic experiences and their impact on proprioception and balance. We attempt to explain how changes in muscle tone and impairments in proprioceptive functioning due to psychological trauma can contribute to an impairment in balance abilities and, thus, an increased injury risk. Looking at these physical manifestations of trauma goes hand in hand with a greater understanding of PTSD beyond its psychological symptoms.

## 2. Materials and Methods

### 2.1. Sample Size Calculation

The required sample size for a 2 (group) multivariate analysis of variance (MANOVA) was determined using G* Power software (https://www.calculator.net/sample-size-calculator.html) [22]. The power analysis was conducted using the following parameters: test type (a) F tests, (b) MANOVA, and (c) between-subject factors, with the input values including a medium effect size (Cohen’s f = 0.25), significance level α = 0.05, and desired statistical power (1 − β) = 0.80. This calculation indicated a minimum required sample size of n = 39.

### 2.2. Participants

We enrolled 48 participants in the study through a multifaceted recruitment approach (13 males and 35 females). The mean age was 20.88 years (*SD* = 1.41). None of the participants had a congenital musculoskeletal disorder, 85% had completed secondary school education, while 2% had an undergraduate degree and 13% had a postgraduate degree. Our experimental initiative was advertised across the Hungarian university’s online social media communities, and via various offline platforms. It is important to note that in our advertising efforts, we took care to avoid portraying the experiment as trauma or abuse research. Instead, we presented it as stress research to provide a more nuanced and accurate depiction of the study’s focus. Following successful completion, participants were assigned a designated code for identification purposes.

Participants were required to be 18 to 25 years old and willing to engage in an experimental activity involving physical movement. We excluded from this study individuals with neurological or psychiatric conditions. Participants with chronic or acute musculoskeletal injuries or pain were not included. Additionally, individuals with medical conditions that hindered movement or impacted their balance, recognition, or cognitive abilities were excluded from participation, as well as those who were professional athletes. Participants were required to provide written consent acknowledging their participation in the study before taking part in the study. Ethical approval was obtained from the Faculty’s Research Ethics Committee at Eötvös Loránd University (2023/493).

### 2.3. Methods

#### 2.3.1. Questionnaires

##### Difficulties in Emotional Regulation Scale (DERS)

To assess emotional regulation difficulties, participants completed the Hungarian version of the Difficulties in Emotion Regulation Scale (DERS), a validated self-report measure designed to evaluate multiple facets of emotion regulation The DERS consists of 36 items distributed across six subscales: (1) non-acceptance of emotional responses, (2) difficulties engaging in goal-directed behavior, (3) impulse control difficulties, (4) lack of emotional awareness, (5) limited access to emotion regulation strategies, and (6) lack of emotional clarity [23]. The items were rated on a 5-point Likert scale, ranging from 1 (almost never) to 5 (almost always), with higher scores indicating more severe emotion regulation difficulties. The Hungarian adaptation has been validated, showing good psychometric properties, including internal consistency and reliability across the subscales [24]. Total scores and subscale scores were calculated for each participant to provide a detailed profile of their emotional regulation challenges.

##### Dissociative Experience Scale II (DES-II)

The Dissociative Experiences Scale II (DES-II) functions as an extensive instrument designed to evaluate a broad spectrum of dissociative experiences, encompassing both normative instances of dissociation, such as daydreaming, and potentially problematic dissociative occurrences [25]. This tool serves primarily as a screening instrument for dissociative disorders, specifically targeting conditions like Dissociative Identity Disorder (formerly known as Multiple Personality Disorder) and other specified dissociative disorders. Notably, individuals diagnosed with Post-Traumatic Stress Disorder tend to score higher on this scale. As an updated version of its predecessor, the DES-II offers improved user-friendliness in its scoring methods. It functions as a self-assessment tool, offering an initial evaluation to determine the potential need for a full clinical interview for dissociative disorders. Furthermore, the scale has been adapted and translated into numerous languages, demonstrating its widespread applicability and usefulness across diverse cultural contexts. Importantly, the authors of the scale have granted permission for its replication in both research and clinical applications. DES-II is readily available for personal use and serves as a key tool in the preliminary assessment of dissociative experiences. The 28-item self-administered questionnaire is designed to measure the prevalence of dissociative symptoms in daily life. Respondents rate the frequency of each experience using a ten-point scale, typically ranging from 0 to 100%. This scale is commonly used in assessments of both healthy and clinical samples.

##### Childhood Trauma Questionnaire

The Brief Childhood Trauma Questionnaire has been specifically designed to examine five distinct categories of maltreatment and neglect experienced during both childhood and adolescence [26]. Comprising 28 items, the questionnaire is divided into five scales: sexual abuse (SA), physical abuse (PA), emotional abuse (EA), physical neglect (PN), and emotional neglect (EN). In addition, two supplementary items are used to evaluate tendencies to downplay or deny instances of abuse, collectively forming a validity scale. The significance of the validity scale is highlighted not only by the questionnaire’s developers but also by various experts in the field. This recognition arises from the crucial role these scales play in assessing the accuracy of trauma reporting and identifying instances of underreporting, thus reducing potential biases in the categorization of the severity of maltreatment and neglect.

#### 2.3.2. OASIS-Pictures

The Open Affective Standardized Image Set (OASIS) was used to evaluate emotional arousal and valence levels in participants. OASIS is a validated collection of 900 images specifically designed for affective research, featuring standardized ratings for valence (positive or negative emotional response) and arousal (intensity of emotional response) [27]. Each image is rated on a 7-point scale for both valence (ranging from unpleasant to pleasant) and arousal (ranging from calm to excited). Participants were shown a subset of 36 images from the OASIS set, selected to represent a wide range of emotional content. The images were categorized based on valence and arousal ratings into three groups: highly positive, moderate, and low levels. The difference in deflections compared to neutral images was analyzed across all output indicators of the balance plate for the entire sample.

#### 2.3.3. Balance Plate

A BTrackS balance plate (Balance Tracking Systems, Inc., San Diego, CA, USA) was utilized to measure postural sway, serving as an objective indicator of physiological responses during the emotional picture presentation. The BTrackS balance plate is a validated force platform that records center of pressure (CoP) data at a sampling rate of 25 Hz, providing real-time measurements of participants’ balance and sway patterns [28]. Participants were instructed to stand barefoot on the balance plate with their feet shoulder-width apart, maintaining a comfortable and stable posture while viewing the images. CoP data were collected continuously throughout the image presentation to assess subtle postural shifts that might correlate with emotional arousal and valence responses. Each trial involved participants standing on the balance plate while viewing the images, enabling a synchronized analysis of postural sway and emotional reactivity. Data processing and analysis were performed using proprietary BTrackS software to calculate total sway, excursion on a mediolateral and anteroposterior angle, RMS (root mean square, indicating the average sway amplitude from the center of pressure), and sway area (ellipse) for each condition. California FDB licensed (#73881), class 1 FDA Medical Device (Balance Tracking Systems, Inc., USA) (#3010668481).

### 2.4. Protocol

**Figure 1:**
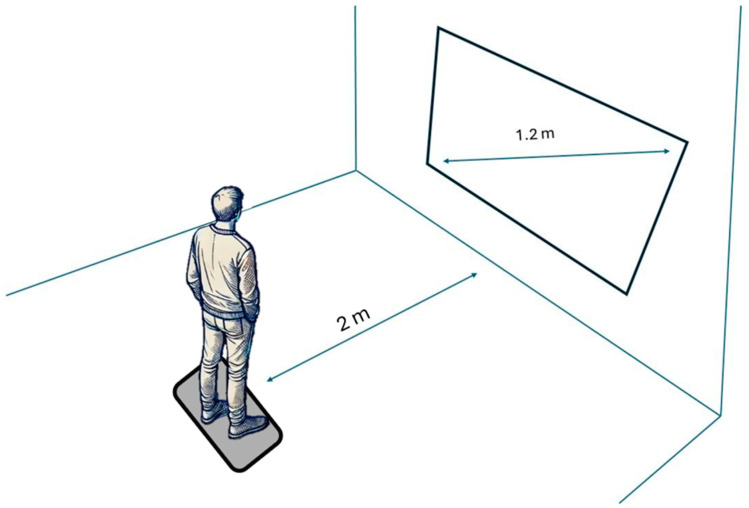
The experimental setup shows a participant standing on the balance plate, positioned two meters from a screen displaying images from the OASIS database.

Upon completing recruitment, participants attended the study in a controlled laboratory setting. Upon arrival, they first filled out a series of questionnaires provided via an online platform on a laboratory laptop. Once they completed the questionnaires, participants were escorted to a separate room where the experimental setup had been prearranged. For details on the experiment room layout, see Figure 1. In this room, they viewed a series of images, which were presented in a randomized order. Each image was displayed for 6 s, with a white screen shown for 2 s between images to avoid carryover effects. During the presentation, participants stood on a BTrackS balance plate, positioned 2 m from the screen displaying the images. The balance plate continuously recorded postural sway as a physiological marker during image viewing. To minimize potential confounding factors, the laboratory environment was controlled for external stimuli such as noise and lighting, ensuring that distractions or disturbances would not influence the participants’ responses.

### 2.5. Statistical Analysis

Statistical analyses were conducted using JASP version 0.18.3. Normality was assessed with the Shapiro–Wilk test, which revealed a non-normal distribution. Consequently, group comparisons were performed using the Mann–Whitney U test. Correlation analyses were carried out using Kendall’s Tau, and reliability was evaluated with Cronbach’s alpha.

## 3. Results

Although emotion regulation skills did not affect participants’ postural sway, some outcomes have been affected significantly by dissociative experience, physical abuse, and physical neglect.

### 3.1. Descriptive Statistics of the Questionnaires

The consent form and questionnaires, including the Emotion Regulation Scale, Dissociative Experience Scale II, and Childhood Trauma Questionnaire were completed by 53 participants. However, due to technical issues, only 48 of them had balance plate data, and only their results are reported in this article. For descriptive statistics of each questionnaire see Table 1.

### 3.2. Effect of Emotion Regulation Skills on Postural Sway

The total score of the Emotion Regulation Questionnaire for the entire sample did not correlate significantly with any of the balance plate variables, neither for positive nor for negative images. Similarly, none of the subscales showed a significant correlation with either positive or negative images with balance plate outcomes. (For correlations, see Table 2, and for descriptive statistics of balance plate outcomes see Table 3).

### 3.3. Effect of Dissociative Experience on Postural Sway

When positive pictures were presented, the number of dissociative experiences did not significantly correlate with total sway, sway area, Excursion ML, or Excursion AP. However, a significant correlation was found with RMS ML.

**Figure 2:**
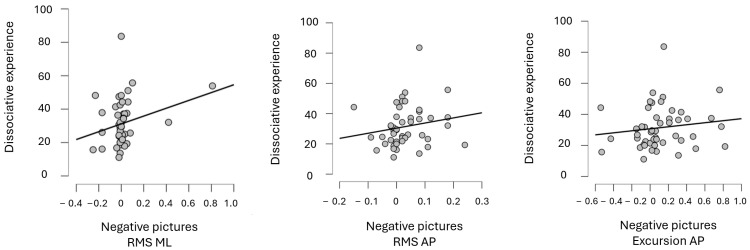
Significant correlations between dissociative experience and balance plate outcomes. Note: RMS (root mean square); ML (mediolateral); AP (antero-posterior).

When negative images were presented to participants, no significant correlations were found for total sway, sway area, or Excursion AP, but significant correlations were observed for RMS ML, RMS AP, and Excursion ML. (For correlations, see Table 4 and Figure 2).

### 3.4. The Effect of Childhood Trauma Experience

We examined whether childhood trauma, specifically childhood abuse, influenced the balance plate deflections when the images were presented. No significant differences were found in any balance plate outcomes between participants with a history of sexual abuse and those without, either during the presentation of positive images or negative images.

Experience of physical abuse had no effect on certain balance plate indicators when viewing positive images but did significantly affect sway area, RMS AP, and Excursion ML. No significant effects were found when viewing negative images.

No significant differences were observed in balance plate outcomes between participants with and without emotional abuse experience when viewing positive images or negative images.

Physical neglect experience did not significantly affect balance plate outcomes when viewing positive images, and did not significantly affect some of the variables while viewing negative pictures. However, it did significantly affect the sway area, RMS AP, Excursion ML, and Excursion AP.

**Figure 3:**
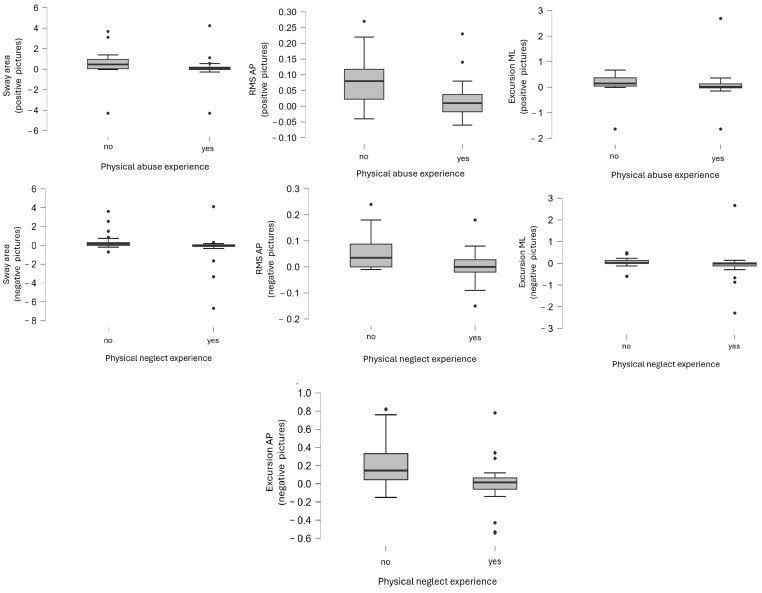
Significant effects of childhood trauma experience on postural sway.

**Figure 4:**
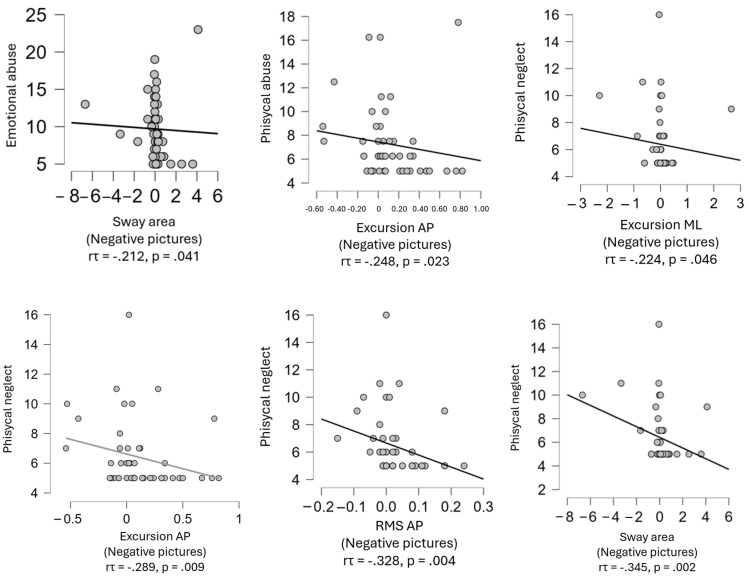
Correlation analysis between abusive experiences and balance plate variables showed a statistically significant relationship, including emotional abuse, physical abuse and physical neglect. Note: RMS (root mean square); ML (mediolateral); AP (anteroposterior).

There was no significant difference in any of the balance plate outcomes between participants who experienced emotional neglect in childhood compared to those who did not, either when watching positive images or negative images. (For test statistics see Table 5 and Figure 3, for descriptive statistics see Table 6 and Figure 4).

## 4. Discussion

This study investigates the impact of trauma on postural control and balance by examining postural sway in response to both neutral and emotionally evocative images. We used images designed to impose arousal and valence in participants, while simultaneously recording their postural sway with a balance plate. Our findings extend the knowledge of the complex interplay between trauma and postural dynamics and thus provide insights into the physical manifestations of Adverse Childhood Experiences and trauma.

Descriptive statistics were first conducted to examine the frequency of Adverse Childhood Experiences (ACEs) within the sample. The Childhood Trauma Questionnaire revealed that emotional neglect had the highest average score, followed by emotional abuse, aligning with patterns frequently observed in trauma research [26]. Consistent with previous studies, emotional neglect and abuse were the most prevalent forms of trauma experienced by participants [29]. In contrast, the average scores for physical and sexual abuse were lower compared to emotional forms of trauma, aligning with ACE literature, where emotional and physical neglect are more common than sexual [30].

Childhood trauma experiences, including those involving physical abuse, influenced certain balance indicators. Although a negative correlation between trauma avoidance and heightened arousal was identified, several other correlations, including those with postural sway, did not reach statistical significance. Results from the Emotion Regulation Scale revealed significant challenges, particularly in limited access to emotion regulation strategies. Our data extend previous findings by indicating that trauma’s effects on motor functioning may be moderated by emotional dysregulation. Specifically, the restricted availability of emotion regulation strategies, as measured by the Difficulties of Emotion Regulation Scale, emerged as a shared challenge in our sample. This suggests that individuals with trauma backgrounds may struggle to manage emotional arousal adaptively. Such dysregulation may also contribute to the observed dissociative patterns and disturbed motor inhibition, where trauma survivors experience a disconnect between emotional states and bodily responses.

Correlation analyses identified significant but selective associations between trauma and various psychological and physical outcomes. Dissociation emerged as a key factor influencing balance, particularly when emotional stressors were present. Specifically, higher dissociation scores were associated with increased mediolateral sway while viewing negative images, suggesting potential motor dysregulation linked to trauma-related dissociative states. This finding supports earlier research indicating that dissociation disrupts sensory-motor integration, leading to altered physical responses during stressful situations [31]. The significant relationship between dissociative experiences and increased sway during negative image exposure highlights how dissociation may impact the body’s ability to regulate posture under stress [32]. These dissociative patterns are commonly associated with PTSD symptoms, whereby excessive arousal leads to maladaptive freeze reactions [33].

The hypothesized relationship between childhood trauma (particularly physical and emotional abuse) and general postural control was weak but statistically significant. This finding regarding postural control aligns with the inconsistencies in the literature related to trauma’s limited direct effects on physical balance measures, and also raises questions about whether the study limitation would provide a statistically lower relationship. Indeed, studies reported similar findings, noting that trauma-related body changes are more apparent during instances of acute stress or when individuals suffer from PTSD. These studies also highlighted the role of increased arousal and hypervigilance in response to trauma triggers, which can either suppress or exaggerate motor outputs depending on the contextual background, in which this study does not control the participants’ arousal or valence level [34].

One possible explanation is given by embodiment theory, which suggests that trauma is stored in the body and can be manifested through posture and motor control [35]. Similarly, the Polyvagal theory, particularly the dorsal vagal branch, postulates that shutdown responses from trauma affects motor regulation [36]. While direct scientific evidence linking either of these theories to balance is limited, our results would suggest that certain types of trauma may influence physical functioning in the ways described by these theories. Considering that some of the results were not significant, several aspects might hint at the reasons behind such findings. One of them could be that postural control is not as sensitive to the effects of long-term trauma as some physiological or psychological measures are when not exposed to acute stressors. Prior research suggests that trauma impacts are more likely to manifest under conditions of heightened arousal or when individuals are triggered by trauma-related stimuli [33]. Our study, however, assessed postural sway in a relatively controlled setting, which may not have fully activated trauma-related physiological responses.

Another possibility is the heterogeneity in traumatic experiences within our sample. Different types of traumas, such as emotional compared to physical abuse, may affect balance control differently. Thus, while emotional abuse/neglect may induce chronic stress responses subtly affecting the nervous system, physical abuse might impact motor control more directly due to the physical nature of the traumatic events. This variability might lead to a diminishment in the findings of significance when analyzing the broad category of trauma [29]. More dynamic measures of motor functioning or stress-reactive tests may uncover aspects of how trauma impacts the body that are not possible to discover under the conditions of this study, as research into trauma-induced motor freezing suggests [34].

The relationship between the freezing reaction and musculoskeletal injuries warrants careful consideration. While the freezing response serves as a protective mechanism in high-stress situations, its chronic activation can lead to negative physical outcomes. Prolonged immobilization during freezing can result in muscle stiffness, reduced range of motion, and altered biomechanics, all of which contribute to an increased risk of musculoskeletal injuries. Furthermore, the associated muscle tension and motor control dysregulation may exacerbate pre-existing conditions, leading to a cycle of pain and injury. This highlights the need for a multidisciplinary approach in addressing stress-related injuries, incorporating strategies to manage both the psychological and physical aspects of the freezing response. Understanding this complex interplay is essential for developing effective rehabilitation and prevention strategies to mitigate the long-term consequences of stress-induced motor inhibition on musculoskeletal health. Drawing from the insights derived from our results, rehabilitation should include specific interventions aimed at improving emotional regulation and proprioceptive awareness tailored to the trauma type. Understanding these physical effects has significant implications for the treatment of trauma. Integrating proprioceptive and balance training into therapeutic interventions could help trauma survivors regain control of their bodily movements, reduce injury risk, and improve overall quality of life [6]. Freezing, a common response to trauma, can also contribute to an increased risk of physical injury due to impaired motor coordination and reduced awareness of bodily movements [12]. Prolonged freeze responses may lead to muscle stiffness and compromised balance, making individuals more susceptible to falls or other injuries [37]. Incorporating movement-based therapies that focus on proprioception and balance could mitigate these risks by enhancing bodily awareness and fostering resilience in trauma survivors.

Further research should explore the deeper interaction and possible mechanisms between emotional regulation deficits, dissociation, and trauma in postural control, and specifically on how certain types of trauma might necessitate different therapeutic approaches to effectively address these challenges to understand and offer insights into musculoskeletal injury prevention and possible therapeutic options for trauma-induced psychological disorders.

### Limitation

One limitation of our study is that we did not record participants’ subjective emotional responses during the picture presentation. While we conducted a brief interview afterwards to gather information about their emotional experiences, real-time emotional ratings during the task would have provided valuable data for comparing subjective emotions with postural changes. This could have offered deeper insights into the relationship between emotional arousal and physiological responses. Additionally, although we excluded participants who were athletes, our sample still included individuals who might be more physically active than the average Hungarian population of the same age group. As a result, these participants may have had superior proprioceptive abilities and balance, which could have influenced their postural stability during the task. Another important limitation is the relatively small number of participants, which may reduce the accuracy of the results. This limits the generalizability of our findings, as the postural sway patterns observed may not fully represent those of the general population. Future studies should aim to recruit a more representative sample to better understand the influence of physical activity levels on balance and emotional regulation.

## 5. Conclusions

This study addresses the relationship between balance, traumatic experiences, and potential musculoskeletal injury prevention. An experimental study was conducted using a balance plate to examine how trauma influences postural control. Our results show that dissociation significantly affects balance, particularly in response to negative emotional stimuli. We hypothesize that traumatic experiences, particularly in childhood, may disrupt postural stability by inducing emotional dysregulation, with statistically significant effects observed under experimental conditions. However, the associations between childhood trauma and balance were weaker than anticipated, suggesting that the impact may vary depending on the specific type of trauma. Limitations in sample size, diversity, and experimental controls may have influenced these outcomes. Thus, further research is necessary to fully understand the complexity of this phenomenon, and the result of the present study should be interpreted with caution. This work emphasizes the need for a deeper investigation into the effects of trauma on postural control and highlights the increased musculoskeletal injury risk in populations with a history of trauma.

## Figures and Tables

**Table 1:** Descriptive statistics for each questionnaire.

Questionnaires (*n* = 48)	*Mean M (SD)*	*Mdn*
**Emotion Regulation Scale**		
Non-Acceptance of Emotional Responses	14.38 (6.03)	12.50
Difficulties Engaging in Goal-Directed Behavior	15.67 (4.68)	16.50
Impulse Control Difficulties	14.83 (5.40)	15.00
Lack of Emotional Awareness	14.29 (5.11)	13.50
Limited Access to Emotion Regulation Strategies	18.68 (7.03)	18.50
Lack of Emotional Clarity	11.40 (3.67)	11.00
Total score	89.54 (24.27)	87.50
**Dissociative Experience Scale II**		
Total core	31.43 (13.65)	28.95
**Childhood Trauma Questionnaire**		
Sexual Abuse	6.15 (2.72)	5.00
Physical Abuse	7.27 (3.10)	6.25
Emotional Abuse	9.67 (4.22)	9.00
Physical Neglect	6.40 (2.28)	5.00
Emotional Neglect	11.25 (3.45)	12.50

**Table 2:** Correlation between Emotion Regulation Questionnaire and balance plate outcomes.

Emotion Regulation Questionnaire Scales/Balance Plate Outcomes	*rτ*	*p*-Value	*rτ*	*p*-Value
	Positive Images		Negative Images	
**Total Score**				
Total Sway	0.073	0.466	0.061	0.575
Sway Area	0.073	0.466	0.098	0.332
RMS ML	0.060	0.560	0.141	0.175
RMS AP	0.056	0.586	0.057	0.579
Excursion ML	0.061	0.551	0.121	0.232
Excursion AP	0.042	0.676	0.048	0.637
**Non-Acceptance of Emotional Responses**				
Total Sway	0.002	0.986	−0.011	0.915
Sway Area	0.022	0.830	0.078	0.448
RMS ML	−0.010	0.921	0.083	0.433
RMS AP	−0.040	0.700	−0.003	0.979
Excursion ML	0.011	0.915	0.093	0.371
Excursion AP	0.007	0.943	−0.026	0.803
**Difficulties Engaging in Goal-Directed Behavior**				
Total Sway	0.073	0.466	0.061	0.575
Sway Area	0.073	0.466	0.098	0.332
RMS ML	0.060	0.560	0.141	0.175
RMS AP	0.056	0.586	0.057	0.579
Excursion ML	0.061	0.551	0.121	0.232
Excursion AP	0.042	0.676	0.048	0.637
**Impulse Control Difficulties**				
Total Sway	0.074	0.470	0.121	0.235
Sway Area	0.058	0.574	0.123	0.232
RMS ML	0.035	0.740	0.139	0.189
RMS AP	0.056	0.591	0.111	0.290
Excursion ML	0.029	0.782	0.126	0.224
Excursion AP	0.025	0.810	0.107	0.296
**Lack of Emotional Awareness**				
Total Sway	−0.089	0.387	−0.069	0.503
Sway Area	0.076	0.459	0.035	0.734
RMS ML	0.064	0.547	0.115	0.279
RMS AP	0.052	0.616	−0.004	0.971
Excursion ML	0.068	0.514	0.052	0.615
Excursion AP	−0.032	0.755	−0.030	0.786
**Limited Access to Emotion Regulation Strategies**				
Total Sway	0.097	0.340	0.077	0.449
Sway Area	0.058	0.568	0.080	0.433
RMS ML	0.054	0.609	0.098	0.350
RMS AP	0.028	0.789	0.043	0.680
Excursion ML	0.043	0.675	0.094	0.362
Excursion AP	0.033	0.748	0.073	0.476
**Lack of Emotional Clarity**				
Total Sway	0.094	0.632	0.052	0.617
Sway Area	0.188	0.068	0.134	0.195
RMS ML	0.160	0.133	0.167	0.119
RMS AP	0.128	0.223	0.108	0.310
Excursion ML	0.154	0.140	0.117	0.263
Excursion AP	0.109	0.291	0.103	0.321

**Table 3:** Descriptive statistics of all the balance plate variables.

	Total Sway (*n* = 48)		Sway Area (*n* = 48)		RMS ML (*n* = 48)		RMS AP (*n* = 48)		Excursion ML (*n* = 48)		Excursion AP (*n* = 48)	
	Mean (*SD*)	*Mdn*	Mean (*SD*)	Mdn	Mean (*SD*)	*Mdn*	Mean (*SD*)	*Mdn*	Mean (*SD*)	*Mdn*	Mean (SD)	Mdn
Positive pictures	0.625 (1.693)	0.620	0.277 (1.339)	0.125	−0.218 (1.729)	0.010	0.041 (0.077)	0.020	0.099 (0.549)	0.045	0.022 (0.910)	0.090
Negative pictures	0.192 (1.524)	0.240	0.046 (1.463)	0.015	0.011 (0.151)	0.000	0.030 (0.072)	0.020	−0.019 (0.566)	0.010	0.112 (0.291)	0.060

**Table 4:** Effect of dissociative experience on balance plate outcomes.

Balance Plate Outcomes	Positive Images		Negative Images	
	*rτ*	*p*	*rτ*	*p*
Total Sway	0.052	0.600	0.168	0.093
Sway Area	0.147	0.142	0.171	0.088
RMS ML	0.207	0.045 *	0.204	0.049 *
RMS AP	0.083	0.417	0.209	0.042 *
Excursion ML	0.123	0.226	0.200	0.049 *
Excursion AP	0.052	0.606	0.142	0.157

note. * = *p* < 0.05.

**Table 5:** The effect of trauma experience on postural sway.

Childhood Trauma Experience /Balance Plate Outcomes	Positive Images		Negative Images	
	*U*	*p*	*U*	*p*
**Sexual Abuse**				
Total Sway	222.50	0.650	208.50	0.912
Sway Area	233.50	0.469	268.00	0.116
RMS ML	192.00	0.786	253.50	0.221
RMS AP	245.50	0.308	224.00	0.662
Excursion ML	210.50	0.873	273.00	0.090
Excursion AP	210.50	0.873	188.50	0.722
**Physical Abuse**				
Total Sway	336.50	0.160	261.50	0.865
Sway Area	374.50	0.027 *	325.50	0.241
RMS ML	340.50	0.134	317.00	0.318
RMS AP	383.50	0.016 *	334.00	0.116
Excursion ML	397.00	0.007 *	329.50	0.208
Excursion AP	350.50	0.088	362.50	0.051
**Emotional Abuse**				
Total Sway	154.00	0.579	204.00	0.459
Sway Area	210.50	0.362	244.00	0.072
RMS ML	172.00	0.936	237.50	0.101
RMS AP	208.50	0.390	216.00	0.288
Excursion ML	189.50	0.721	214.00	0.315
Excursion AP	210.50	0.362	217.00	0.279
**Physical Neglect**				
Total Sway	361.00	0.123	367.00	0.096
Sway Area	366.50	0.098	428.00	0.003 *
RMS ML	314.50	0.560	359.50	0.128
RMS AP	371.00	0.080	424.50	0.004 *
Excursion ML	347.00	0.210	388.50	0.034 *
Excursion AP	353.00	0.169	411.50	0.010 *
**Emotional Neglect**				
Total Sway	90.00	0.566	128.00	0.500
Sway Area	86.50	0.489	118.00	0.723
RMS ML	78.00	0.325	102.50	0.865
RMS AP	79.50	0.352	91.50	0.599
Excursion ML	53.50	0.071	87.50	0.510
Excursion AP	82.50	0.408	88.00	0.521

note. * = *p* < 0.05.

**Table 6:** Descriptive Statistics of Balance Plate Variables in Childhood Traumatic Experiences.

	Positive Pictures								Negative Pictures							
	Traumatized				Non-Traumatized				Traumatized				Non-Traumatized			
	*Mean*	*SD*	*Mdn*	*n*	*Mean*	*SD*	*Mdn*	*n*	*Mean*	*SD*	*Mdn*	*n*	*Mean*	*SD*	*Mdn*	*n*
**Sexual Abuse**																
Total Sway	0.589	0.709	0.610	11	0.635	1.898	0.630	37	0.055	0.916	0.210	11	0.233	1.671	0.240	37
Sway Area	0.215	0.452	0.090	11	0.295	1.510	0.130	37	−0.348	1.025	−0.060	11	0.164	1.562	0.030	37
RMS ML	0.027	0.044	0.010	11	−0.277	1.969	0.010	37	−0.033	0.074	−0.010	11	0.024	0.166	0.000	37
RMS AP	0.012	0.040	0.010	11	0.049	0.084	0.030	37	0.014	0.030	0.020	11	0.035	0.080	0.020	37
Excursion M	0.117	0.187	0.040	11	0.094	0.620	0.050	37	−0.133	0.278	−0.010	11	0.014	0.626	0.010	37
Excursion AP	0.107	0.198	0.080	11	−0.003	1.034	0.110	37	0.101	0.120	0.070	11	0.115	0.326	0.060	37
**Physical Abuse**																
Total Sway	0.537	1.652	0.590	30	0.771	1.798	0.705	18	0.147	1.710	0.255	30	0.267	1.193	0.080	18
Sway Area	0.117	1.155	0.090	30	0.543	1.600	0.460	18	−0.124	1.499	0.025	30	0.331	1.394	0.005	18
RMS ML	0.045	0.177	0.010	30	−0.628	2.812	0.030	18	0.019	0.184	0.000	30	−0.002	0.069	0.000	18
RMS AP	0.018	0.057	0.010	30	0.078	0.092	0.080	18	0.012	0.061	0.015	30	0.061	0.080	0.045	18
Excursion M	0.082	0.592	0.015	30	0.127	0.485	0.145	18	−0.042	0.686	0.005	30	0.018	0.284	0.015	18
Excursion AP	0.074	0.230	0.040	30	−0.064	1.479	0.180	18	0.031	0.255	0.035	30	0.247	0.302	0.225	18
**Emotional Abuse**																
Total Sway	0.734	1.520	0.600	39	0.149	2.358	0.630	9	0.133	1.592	0.210	39	0.449	1.230	0.450	9
Sway Area	0.225	1.051	0.190	39	0.503	2.282	0.110	9	−0.147	1.433	0.000	39	0.883	1.359	0.160	9
RMS ML	0.047	0.156	0.010	39	−1.309	3.970	0.010	9	0.007	0.166	0.000	39	0.027	0.042	0.010	9
RMS AP	0.033	0.068	0.070	39	0.074	0.107	0.020	9	0.022	0.064	0.010	39	0.066	0.094	0.050	9
Excursion M	0.119	0.527	0.130	39	0.012	0.667	0.040	9	−0.048	0.618	0.010	39	0.104	0.221	0.020	9
Excursion AP	−0.031	0.985	0.190	39	0.254	0.434	0.080	9	0.077	0.265	0.050	39	0.262	0.363	0.210	9
**Physical Neglect**																
Total Sway	0.716	1.490	0.450	22	0.547	1.874	0.755	26	−0.032	1.986	0.095	22	0.382	0.986	0.430	26
Sway Area	0.344	0.956	0.070	22	0.220	1.610	0.195	26	−0.362	1.865	−0.055	22	0.392	0.912	0.155	26
RMS ML	0.067	0.192	0.010	22	−0.440	2.338	0.015	26	0.022	0.216	−0.010	22	0.002	0.059	0.000	26
RMS AP	0.018	0.062	0.005	22	0.060	0.084	0.025	26	−0.002	0.064	0.000	22	0.057	0.068	0.035	26
Excursion M	0.175	0.578	0.030	22	0.035	0.526	0.130	26	−0.075	0.804	−0.010	22	0.028	0.234	0.020	26
Excursion AP	0.096	0.254	0.010	22	−0.040	1.223	0.165	26	−0.003	0.279	0.015	22	0.209	0.268	0.145	26
**Emotional Neglect**																
Total Sway	0.653	1.779	0.610	43	0.376	0.614	0.640	5	0.180	1.604	0.210	43	0.294	0.501	0.450	5
Sway Area	0.300	1.414	0.130	43	0.074	0.118	0.110	5	0.044	1.546	0.000	43	0.066	0.171	0.080	5
RMS ML	−0.232	1.827	0.010	43	0.004	0.019	0.010	5	0.013	0.159	0.000	43	−0.004	0.017	0.000	5
RMS AP	0.045	0.080	0.020	43	0.006	0.032	0.010	5	0.032	0.074	0.020	43	0.012	0.048	0.020	5
Excursion M	0.110	0.580	0.050	43	0.002	0.074	−0.020	5	−0.019	0.599	0.010	43	−0.022	0.056	−0.010	5
Excursion AP	0.020	0.962	0.110	43	0.038	0.147	0.040	5	0.120	0.301	0.060	43	0.042	0.192	0.040	5

## Data Availability

The raw data supporting the conclusions of this article will be made available by the authors on request.
